# Review of Applications of Near-Infrared Spectroscopy in Two Rare Disorders with Executive and Neurological Dysfunction: UCD and PKU

**DOI:** 10.3390/genes13101690

**Published:** 2022-09-21

**Authors:** Kosar Khaksari, Wei-Liang Chen, Andrea L. Gropman

**Affiliations:** 1 Division of Neurogenetics and Developmental Pediatrics, Children’s National Health System, Washington, DC 20010, USA; 2 School of Medicine, University of Washington, Seattle, WA 98195, USA; 3 Department of Neurology, George Washington University, Washington, DC 20052, USA

**Keywords:** near-infrared spectroscopy, rare diseases, executive dysfunction, brain biomarkers, urea cycle disorder, phenylketonuria, neuromonitoring, ammonia

## Abstract

Studying rare diseases, particularly those with neurological dysfunction, is a challenge to researchers and healthcare professionals due to their complexity and small population with geographical dispersion. Universal and standardized biomarkers generated by tools such as functional neuroimaging have been forged to collect baseline data as well as treatment effects. However, the cost and heavily infrastructural requirement of those technologies have substantially limited their availability. Thus, developing non-invasive, portable, and inexpensive modalities has become a major focus for both researchers and clinicians. When considering neurological disorders and diseases with executive dysfunction, EEG is the most convenient tool to obtain biomarkers which can correlate the objective severity and clinical observation of these conditions. However, studies have also shown that EEG biomarkers and clinical observations alone are not sensitive enough since not all the patients present classical phenotypical features or EEG evidence of dysfunction. This article reviews disorders, including two rare disorders with neurological dysfunction and the usefulness of functional near-infrared spectroscopy (fNIRS) as a non-invasive optical modality to obtain hemodynamic biomarkers of diseases and for screening and monitoring the disease.

## 1. Introduction

### 1.1. Rare Diseases, Biomarkers, and Diagnostic Methods

Rare diseases are defined as uncommon conditions with a prevalence of less than 50 per 100,000 (1 in 2000) people or less than 200,000 people in the US [1,2,3,4,5]. Even with their rarity, together they are not uncommon. Collectively, 60 million people in the US and EU alone are suffering from rare diseases [6]. Approximately 7000 rare diseases are recognized, and this number is continuing to rise due to advances in genomic technology [3]. Many rare diseases can involve multiple organ systems, and the prognoses and levels of rareness may vary. Some organ systems are more accessible for study by standard technologies (such as echocardiogram for the system); others require more sophisticated approaches to assess their function from a distance (such as functional magnetic resonance imaging or positron emission tomography for the central nervous system). Therefore, in addition to the small population of eligible subjects, geographic dispersion of patients, and the unique characteristics of rare diseases, the accessibility of the affected systems for functional study creates another layer of complexity for the development of biomarker assessment and ultimately targeted treatments for these diseases [7,8]. Among all rare diseases, urea cycle disorder (UCD) and phenylketonuria (PKU), in the context that they are associated with executive dysfunction, constitute the main interest of this work [1,3,5,7].

In recent years, treatments for rare diseases have been advancing substantially, largely due to advances in genomics and biotechnology allowing the development of enzyme replacement therapy, gene therapy and gene-targeted modifications/corrections. However, measuring the neurocognitive and neurological outcomes of disorders associated with altered neural networks remains challenging. For the purpose of outcome measurement, the use of biomarkers or other surrogate endpoints is necessary if current endpoints are not identified for the future patient population [5]. A biological marker or biomarker serves as an objectively measured feature that can be assessed as a signal of normal or pathological progressions. A biomarker, ideally, can detect early symptoms and may be used to monitor disease prognosis. There are numerous platforms for the identification of biomarkers for rare diseases, however, very few of the candidate platforms are validated clinically due to the small population and complications of rare disease studies [5,9,10]. Some work suggests that a group of biomarkers, or a biomarker panel rather than a single biomarker, could be employed for overcoming the challenges associated with some methods [5,11].

Biomarkers can be substances, specific cells or molecules, genes, enzymes, hormones, and signal features that show specific changes in characteristics of biological structures or body tissues [11]. Serum biomarkers have been investigated for their potential to provide measures in the assessment of brain injuries and dysfunction. Among those, no biomarker has had excellent performance, but several demonstrated appropriate correlation, including markers of coagulation and inflammation, structural proteins in the brain, and proteins involved in homeostasis [12]. Translational neuroimaging techniques provide another set of biomarkers for tracking injuries and inflammatory processes in the brain. Electroencephalography (EEG), functional magnetic resonance imaging (fMRI), positron emission tomography (PET), single-photon emission computerized tomography (SPECT), magnetic resonance spectroscopy (MRS), diffusion tensor imaging (DTI), and near-infrared spectroscopy (fNIRS) are imaging modalities that monitor biomarkers of the brain [13]. In this work, we focus on the advantages of fNIRS in the diagnosis, early detection and monitoring the prognosis and recovery of rare diseases. Since fNIRS is a novel neuroimaging modality, there are few studies using this technology for studying rare diseases. This review will discuss the feasibility of using fNIRS in rare diseases with cognitive and executive dysfunction.

### 1.2. fNIRS and Cerebral Hemodynamics

**Figure 1:**
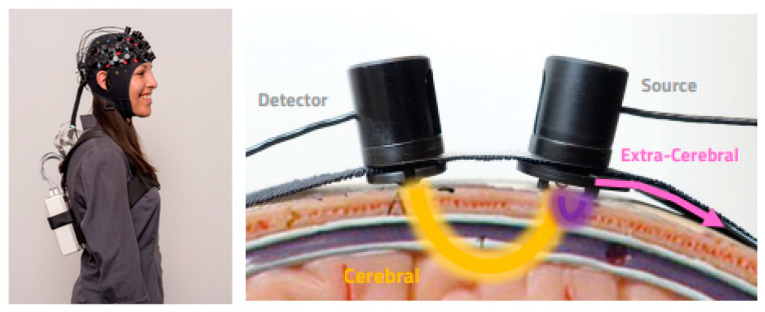
NIRSport2 device from NIRx as a small a backpack (**left**). NIRS source-detector placement (**right**). Photo courtesy: https://nirx.net/ (accessed on 30 March 2021).

Functional near-infrared spectroscopy (fNIRS) is a non-invasive neuroimaging modality that reveals features of brain hemodynamics. It has been used extensively in clinical settings. fNIRS uses near-infrared (IR) light to measure the diffusion of photons in human tissue or cortical tissue in the case of brain imaging. Hemodynamic fluctuations in the cortex are calculated via the difference in light absorbance of oxygenated hemoglobin and deoxygenated hemoglobin. Figure 1 shows the optode placement and the interaction of light with brain tissue in a so-called banana-shaped region. There are other methods such as fMRI, PET and SPECT that similarly monitor cerebral blood flow and volume, each with their own disadvantages. For example, fMRI has been the traditional imaging technique used to study the relationships between patterns of functional brain activation in different neurological diseases. However, fMRI is relatively expensive and is very sensitive to motion. It requires the subject to remain completely still to attain usable data, which makes it an unsuitable case for studying infants, children, those with cognitive impairment and with executive dysfunction.

fNIRS has several unique features and offers advantages to fMRI such as its non-invasiveness, portability, tolerability, low cost, real-time measurement of both hemoglobin species, and the capability to be co-registered with EEG, along with Bluetooth functionality, which make it an ideal choice to study brain biomarkers in certain clinical settings [14,15]. For example, fNIRS has become the standard tool for monitoring cerebral oxygenation in pediatric ICUs [16]. In the case of rare diseases, the portability of fNIRS will allow researchers to travel to the geographic areas where the small eligible populations can be tested. Furthermore, because of relatively low sensitivity to motion, fNIRS can be a particularly useful tool for measurements on children and toddlers with rare diseases who cannot tolerate the fMRI [17]. The limitations in using fNIRS are its low spatial resolution and potential signal contamination from the extracerebral layers, physiological noise, and motion artifacts. New fNIRS technologies and signal processing approaches have been developed to overcome those limitations.

In the present review, we will discuss the use of fNIRS in diseases with neurological dysfunction. Urea cycle disorder (UCD) and phenylketonuria (PKU) will be reviewed, and the outcomes of related studies will be discussed. Among these diseases, fNIRS has been introduced for investigating executive function in UCD [18,19].

## 2. Literature Search

A literature review was performed in Google Scholar, PubMed and ScienceDirect to search for studies regarding rare diseases from 1953 to the present. The following key words were utilized: rare diseases, electroencephalography, EEG, near-infrared spectroscopy, fNIRS, cognitive dysfunction, executive dysfunction, neurological dysfunction, urea cycle disorder, UCD, phenylketonuria, PKU, neuroimaging, brain impairment, brain biomarkers, non-invasive brain imaging, brain signal, and brain hemodynamics. Our main goal was to find, review and evaluate articles that used fNIRS for screening brain functional activity in patients with neurological impairment due to urea cycle disorder and phenylketonuria.

## 3. Rare Diseases

### 3.1. Urea Cycle Disorder (UCD)

The urea cycle disorders (UCD) are a group of genetic disorders associated with an enzyme or a carrier protein insufficiency [20,21]. Table 1 shows the specific UCD and prevalence. Removing ammonia, a neurotoxic substance, from the blood is hindered in the UCD. Accumulation of ammonia, namely, hyperammonia (HA), can cause severe brain damage and encephalopathy in several of the UCD [22]. The cognitive sequelae may range from mild to severe executive dysfunction and working memory impairment to intellectual disability and seizures [23,24]. There are several methods to investigate brain function and structure during HA such as electroencephalography (EEG), magnetic resonance imaging (MRI), diffusion tensor imaging (DTI), magnetic resonance spectroscopy (MRS) and functional near-infrared spectroscopy (fNIRS). These modalities allow for the non-invasive monitoring of disease prognostic, brain-neurological dysfunction and brain-related pathophysiology of UCD. fNIRS offers portability, and immediate availability in a patient who is too hemodynamically unstable to transport to the MRI suite.

One of the main focuses in studying brain function in UCD is to understand the pathogenesis of brain dysfunction and its temporal correlation to the HA. It has been postulated that there is a critical age or stage for HA to cause irreversible dysfunction. It is also important to know if there are differences between UCDs in their mechanism of brain damage [25].

In 2003, the urea cycle disorder consortium (UCDC) was established under the Rare Diseases Clinical Research Network (RDCRN) by the US National Institutes of Health (NIH). Since then, research centers and clinical research to address gaps in treatment and monitoring of UCD patients have grown.

In order to answer the questions relating to brain dysfunction, early efforts were made by the UCDC [26]. Focusing on the mechanisms of neuronal injury secondary to HA, they concluded that the major consequence of UDC is neurological, but it is unclear whether the HA was the only contributor to the pathogenesis. For instance, glutamine could also play a role. Other key studies included measuring urea production in patients with urea cycle defects in vivo [27], a randomized, double-blind, crossover study of sodium phenylbutyrate and low-dose arginine (100 mg/kg/day)compared to high-dose arginine alone on liver function, ureagenesis and subsequent nitric oxide production in patients with argininosuccinate lyase (AL) deficiency [21], and carbamylglutamate treatment of n-acetyl glutamate synthetase deficiency (NAGS) [28,29]. Among these studies, only Gropman et al. considered imaging modalities for investigating the neurological manifestation of the disease [18,30].

**Figure 2:**
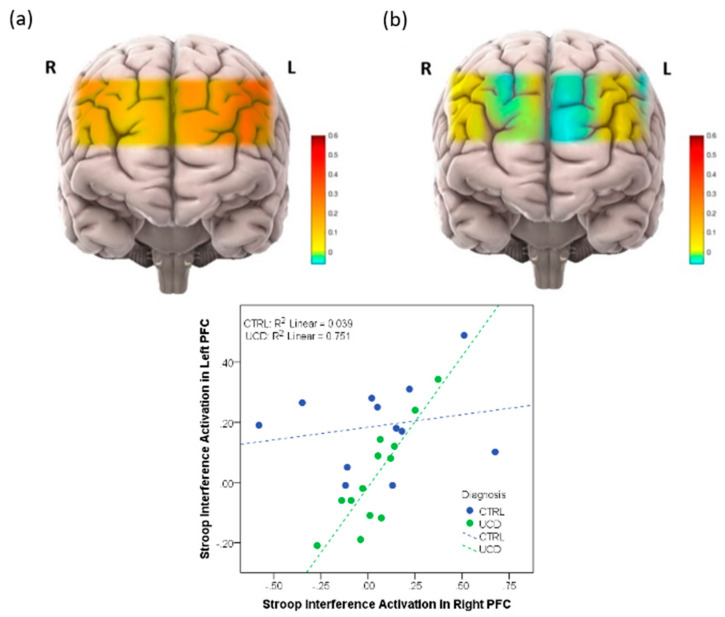
(**Top**): (**a**) Activation map in prefrontal cortex (PFC) in healthy volunteers, (**b**) Activation map in prefrontal cortex in patients with UCD. (**Bottom**): Correlation between right and left PFC in subjects with and without UCD.

As referenced above, neurological impairment is of primary significance to the UCD [13,26]. For decades, EEG and MRI have been the main modalities for studying neurological dysfunction in the UCD [20]. fNIRS has emerged as a non-invasive technique and could serve as a surrogate to fMRI with better portability in assessing hemodynamic change in the brain and further assist the development of new imaging biomarkers [31]. Like other biomarkers, information forms a single modality and can be misleading in a clinical setting due to the inherent limitation of likelihood ratios. Thus, combining neuroimaging modalities has been considered so that the data from multiple imaging modalities can overcome the incomplete information from individual modalities. The portability of fNIRS is particularly important when working with children and toddlers, but also with populations who will have difficulty remaining still in a scanner or have claustrophobia. Multimodal imaging with EEG and fNIRS was introduced as potential real-time imaging for detection and treatment of brain injuries due to UCD and HA. Figure 2 illustrates the activation map and the difference between ornithine transcarbamylase deficiency (OTCD) subjects and normal controls during a Stroop task that resulted from this study. A lower activation during the Stroop task was seen in patients with OTCD [13]. These results confirm the fMRI outcomes in OTCD patients. A significant change in brain activation was also seen in BOLD signal on PFC of OTDC patients using fMRI compared to healthy controls [30].

Two published articles used fNIRS to measure brain activation in UCD patients. Anderson et al. measured brain activation in prefrontal cortex (PFC) on OTCD patients when they performed the Stroop task [18]. In this study, the differences in brain activation in left and right PFC in patients with OTCD and controls was assessed. It was found that control subjects demonstrated a higher task-related activation increase, whereas subjects with OTCD also exhibited bilateral increase in PFC activation. This was in the absence of a significant difference in response time or correct responses. They concluded that fNIRS can provide a unique opportunity to monitor OTCD progression and examine neurocognitive changes. This work confirmed the activation pattern found using fMRI through an N-back task that showed a higher dorsolateral PFC activation in patients with OTCD [30]. The second work from Anderson et al. was a twin study to examine the hemodynamics of PFC in a fraternal twin with and without OTCD [19]. They quantified the hemodynamics of the brain as measured by fNIRS while subjects performed the N-back working memory task. Results demonstrated that the sibling with OTCD showed higher variation in a very low frequency band (related to cerebral autoregulation) compared to the control sibling. The difference between these variations was not as obvious in the higher frequency band, indicating the possible autoregulation impairment and cognitive dysfunction due to the presence of HA. This work was the first to show the efficacy of fNIRS in monitoring patients with UCD and indicated the possibility of inefficient neurocognitive function. Sen et al. in their review paper on UCD research, reviewed these works and mentioned that they believed fNIRS could provide a novel and non-invasive tool to monitor OTCD progression and to examine neurocognitive changes [20]

### 3.2. Phenylketonuria(PKU)

Phenylketonuria (PKU) is a genetic autosomal recessive disorder that causes the amino acid phenylalanine to accumulate in the body. In the United States, the incidence of PKU is approximately 1 in 10,000. Different ethnicities also have different prevalence (1 in 8000 among Caucasians and 1 in 50,000 among African Americans) [32]. The defect that results in PKU is found on chromosome 12 at the locus 12q24.1 [32] and is caused by a defect in the phenylalanine hydroxylase (*PAH*) gene that helps create the enzyme needed to break down phenylalanine. Without the enzyme to process phenylalanine, a dangerous buildup of phenylalanine (Phe) can develop when a person with PKU eats foods that contain protein with high amounts of Phe.

PKU is the first known inherited cause of intellectual disability in pediatrics [33]. Infants in the United States and many other countries are screened for PKU soon after birth. Recognizing PKU early on can help prevent significant health problems. Patients show neurological abnormalities, intellectual disability, poor motor function, microcephaly, epilepsy and pyramidal tract dysfunction [34]. Universal newborn screening pioneered by Dr. Ronald Scott and the introduction of low phenylalanine diet in 1953 by Bickel et al. [35] have made the serious side effects of PKU become a rare incidence.

Although the serious side effects of PKU have been greatly prevented, the subtle or subclinical neurological changes can still be seen, especially in adults with high Phe level [36]. Therefore, the correlation between the Phe level and cortical function is still of great interest for researchers and clinicians. PKU is the best studied inborn error of metabolism about the impact of elevated Phe levels on the brain and its impact on executive function. Several imaging modalities have been used to study PKU, but only some of them targeted the brain functional activity of this population. Thompson et al., (1993) reported MRI findings of 34 subjects [34].

In addition to the structural changes, the functional aspect of the brain has also been explored in individuals with PKU. Christ et al. have looked at brain function in PKU and have done the most in PKU using fMRI [37]. They reported atypical PFC neural activity in PKU patients during N-back working memory tasks. They also tested the PKU-related functional connectivity impairments compared to normal controls and found decreased connectivity within and between the PFC (and other brain regions) in PKU patients. Their results are consistent with previous behavioral analysis which showed the prefrontal dysfunction in this cohort [38,39].

Abgottspon et al. examined the neural activity and working memory performance in individuals with PKU compared to controls using fMRI [40]. Their cohort was early-treated adults with PKU and aged matched normal adult controls. They concluded that cognitive performance and neural activity is altered in PKU patients which is related to metabolic dysfunction due to the disease. They showed a lower prefrontal activation compared to normal controls. A lower accuracy in responses was also seen in patients with PKU during the fMRI task, but the response time was comparable to controls. This indicates the reduced working memory performance in the PKU population.

Sundermann et al., in their 2020 work, tested a Go-No-Go task in young females with PKU using fMRI. Higher incidents of errors and altered activation were also reported by this group. Their results supported the presence of cognitive dysfunction in the PKU cohort [41]. Using fMRI and N-back task, Trepp et al. defined a clinical study of cognitive deficits in PKU as a part of a large study on this cohort [42].

Mentioning the neuroimaging studies on PKU, we presented fNIRS along with fMRI as imaging modalities of choice for studying the brain dysfunction and impairment in this population. fMRI’s limitations, such as sensitivity to motion, made it a challenging method for the study of PKU patients due to their poor motor function. fNIRS, however, can be an ideal modality that provides increased flexibility to investigate neurological dysfunction in PKU due to its uniqueness in terms of wireless functionality, portability, and insensitivity to motion.

## 4. Discussion

Functional neuroimaging has gained substantial attention in recent years. DTI, SPECT, PET, MRS, MRI and fMRI have been used extensively for various brain injuries and impairments [13,43,44]. fMRI has now become a gold standard to identify an eloquent cortical area for surgical planning. A neurovascular coupling mechanism, which describes the increase in cerebral blood flow with the neural activation, is the basis of all the recent advance in functional neuroimaging. Additionally, radioactively labelled tracer can be in a PET scan to target a more specific molecule in the brain (also known as a neuroimaging biomarker) [45]. Here, we reviewed literature regarding the use of imaging modalities in detecting biomarkers of select disorders with neurological complications and suggested how they might complement current strategies for studying brain function in rare disorders to establish biomarkers for clinical trials. Urea cycle disorder and phenylketonuria were the focus of this review article. There were several methods to record biomarkers of these diseases such as genetic investigation, or identifying molecules, enzymes, and signals of the underlying biological processes. Specifically in diseases with neurological and neurodevelopmental impairments, examining the changes in the structure, and hemodynamic signals of the brain in addition to other vital signals could inform early diagnosis and monitor the prognosis of the disease. However, all those current modalities have limited availability due to their infrastructural requirement and high cost. fNIRS, a non-invasive imaging method, has been considered the modality of choice for studying brain function by quantitating cerebral oxy- and deoxy-hemoglobin signals [14,46,47,48].

fNIRS has been used in the realm of neuroscience. In this work, we reviewed its use for UCD and PKU. In UCD, considering that high brain levels of ammonia lead to tissue damage and severe neurological and cognitive manifestation, early detection of brain biomarkers is crucial in treatment and monitoring the prognosis of the disease. fNIRS, however, has not been used in patients with PKU. Abnormal prefrontal cortical activation to memory-related tasks was seen in fMRI. Task-related activation now can be interrogated with fNIRS as well and a potential expansion of its use to the cohort with PKU will be an area to explore in the future.

There are some limitations in using fNIRS to study neurological impairments, however. Rare diseases such as UCD and PKU have very small population, and these patients are geographically dispersed. Performing measurements on these patients requires significant planning and travel. fNIRS has its own limitations, which are mentioned in many studies [14]. fNIRS signals are contaminated with physiological noise and motion artifacts. Several offline and interactive approaches have been proposed for the correction of these contaminations [49,50]. Physiological noise due to cardiac, respiratory and blood pressure fluctuations can cause false positives and must be removed using suitable filters. An autoregressive (AR) filter was suggested by Baker et al. to remove this type of noise since they have specific frequencies [51]. Motion-artifact correction has also been addressed using different methods. Methods such as transient-artifact reduction algorithm (TARA), principal component analysis (PCA), spline interpolation, Kalman filtering, and wavelet filtering have been reported by various research studies. Based on the data and the type of artifact a suitable motion-artifact correction must be used. Brigadoi et al. and Barker et al. have discussed these approaches thoroughly [50,51,52,53].

Ambient light is another interfering factor that can be removed using a proper experimental setup. Having the lights off and/or covering the probe with a dark cloth would be helpful to get a more reasonable data quality with the least interference of room light [54].

Therefore, group averaging of channels is a common practice in helping to get more accurate information of the hemodynamic signals. In addition, principal component analysis and post-processing of the optical signals using motion-artifact removal methods are also crucial. All those requirements of advanced post-processing have added more complexity and limitation of its use, especially in a real-time manner.

In addition to fNIRS limitations, in order to have enough spatial information, multichannel fNIRS is the solution. There are new fNIRS systems available now that are lightweight, self-contained and do not rely on fiber-optic cable connections to the main instrument, which can make multichannel fNIRS more practical for patients who cannot tolerate the heavy weight of the traditional cap. The other limiting factor in NIRS measurements is the interference of hair as a strong absorber for light. In the case particularly of darker hair colors and thicker hair densities, there may not be enough backscattered light coming back to the detector, affecting signal strength and quality. Even with an individual with lighter hair color, the signal quality still heavily depends on the techniques used by the researcher. This issue can be reduced where individual elements in the emitter detector array are removable to clear hair interposed between them and the skin surface, and where individual channels can be adjusted to deliver optimal optical power. Also, the quality of optical data is improved by systems that have multi-gain power control. The different power levels this provides enables optimal optical quality across a range of ages.

Lastly, the fundamental assumption of all the functional neuroimaging modalities, including fNIRS, is that the biological time series is a linear system. In fact, for most cases, this assumption oversimplified the question we tried to answer. The lack of the linear correlation between two time series (say, EEG and fNIRS) might just reflect the fact that the brain is a non-linear dynamic complex with multiple layers of biological interaction taking place at the same time. Hence, non-linear approaches such as dimensionality, entropy, and connectivity, which are now widely used to analyze the EEG signal, should be considered in interrogating the fNIRS signal as well. Nonlinearity in fNIRS is larger than fMRI and, when probing a wider range of stimulus, rate and amplitude, the chance of seeing non-linearities is higher [55,56]. The availability of fNIRS with a higher sampling rate (up to 100 Hz) will allow researchers to identify more data for nonlinear analysis.

## Figures and Tables

**Table 1:** The specific UCD disorders and estimated prevalence [25].

UCD Disorders	Prevalence
Ornithine transcarbamylase deficiency (OTCD)	1:14,000
Argininosuccinate synthase (AS) deficiency (Citrullinemia)	1:57,000
Carbamyl phosphate synthase I (CPSI) deficiency	1:62,000
Argininosuccinate lyase (AL) deficiency (Argininosuccinic aciduria)	1:70,000
Arginase (ARG) deficiency (Argininemia)	1:350,000
N-acetylglutamate synthase (NAGS) deficiency	unknown
Citrullinemia type II (Mitochondrial aspartate/glutamate carrier deficiency-CITR)	1 in 21,000 in Japan
Hyperornithinemia, hyperammonemia, homocitrullinuria (HHH) syndrome (Or mitochondrial ornithine carrier deficiency-ORNT)	unknown

## Data Availability

Not applicable.
